# Development of a Whole Blood Paper-Based Device for Phenylalanine Detection in the Context of PKU Therapy Monitoring

**DOI:** 10.3390/mi7020028

**Published:** 2016-02-15

**Authors:** Robert Robinson, Liam Wong, Raymond J. Monnat, Elain Fu

**Affiliations:** 1 School of Chemical, Biological, and Environmental Engineering, Oregon State University, Corvallis, OR 97331, USA; robinrob@oregonstate.edu (R.R.); wonglia@oregonstate.edu (L.W.); 2 Departments of Pathology and Genome Sciences, University of Washington, Seattle, WA 98195, USA; monnat@u.washington.edu

**Keywords:** paper-based device, whole-blood assay, phenylalanine detection, colorimetric readout, phenylketonuria

## Abstract

Laboratory-based testing does not allow for the sufficiently rapid return of data to enable optimal therapeutic monitoring of patients with metabolic diseases such as phenylketonuria (PKU). The typical turn-around time of several days for current laboratory-based testing is too slow to be practically useful for effective monitoring or optimizing therapy. This report describes the development of a rapid, paper-based, point-of-care device for phenylalanine detection using a small volume (40 μL) of whole blood. The quantitative resolution and reproducibility of this device with instrumented readout are described, together with the potential use of this device for point-of-care monitoring by PKU patients.

## 1. Introduction

The therapy of many metabolic diseases requires continuous patient monitoring [1]. People with the genetic disorder phenylketonuria (PKU) struggle with the lifelong challenge of maintaining restricted blood phenylalanine (Phe) levels in order to avoid severe neurological sequelae. For PKU patients, the most common treatment is nutritional therapy. Patients are maintained on a diet that is low in Phe and supplemented with synthetic protein formula [2,3,4]. Guidelines from PKU experts at the 2012 National Institutes of Health (NIH) Phenylketonuria Scientific Review Conference recommend that all PKU patients maintain blood phenylalanine levels between 2 and 6 mg/dL throughout life [4]. The substantial person-to-person variation in phenylalanine metabolism among PKU patients [5] means that implementing and maintaining nutritional therapy can be a lengthy and difficult process [2,6,7]. An optimal formula prescription and diet must balance the patient’s age-dependent nutritional requirements for proper development, while restricting blood phenylalanine levels [7]. This is complicated by the fact that an individual’s tolerance for phenylalanine can vary significantly during their lifetime [7], and that PKU patients can face challenges with maintaining a strict therapy regimen at various stages of their life. Maintaining optimal nutritional therapy can be especially challenging for young children, adolescents, and women during pregnancy [2,6]. In addition to nutrition-based treatment, there is one medication-based treatment with a Phe metabolic co-factor that is appropriate for a subset of PKU patients [8], and additional promising treatments are in development [9]. In all of these cases, measuring patient blood Phe levels plays a critical role in implementing and assessing the efficacy of a therapeutic regimen [10].

Current monitoring for PKU patients in the USA consists of sending blood spot samples dried on filter paper for laboratory-based phenylalanine measurement (often via mass spectrometry), which can be supplemented at the time of clinic visits with a venipuncture blood sample for laboratory-based analysis (which can be made by mass spectrometry, high-pressure liquid chromatography, or amino acid analyzer) [10]. The time to result for both of these standard-of-care monitoring approaches is often several days and this time lag represents a critical barrier to improving therapy for many PKU patients. Rapid feedback of Phe levels would enable a PKU patient to sample their blood level at the recommended 2–3 h after a meal [4] and use the information to effectively guide their therapy in near real-time. Further, an inexpensive and simple-to-use field test would enable an increased frequency of testing by decoupling blood sampling from a clinic visit or from having to collect and mail blood spot samples. Specific potential advantages of regular phenylalanine monitoring include improved therapy compliance from timely feedback on patient actions [2] and an increased flexibility in dietary options [6]. The utility of a phenylalanine test for direct use by PKU patients is well-established [6,11,12,13] with PKU patients broadly indicating an interest in being able to perform blood phenylalanine monitoring with increased frequency [2]. The ultimate potential of such a test would be to enable a PKU patient to have a personalized therapy regimen with individually-optimized diet and prescriptions for nutritional supplements and for medication.

Paper microfluidics is a rapidly growing subfield of microfluidics that makes use of paper-like porous materials to create devices for use in low-resource settings such as in the home or remote locations (we use the term “paper” to broadly encompass all porous materials traditionally used in the lateral flow industry). Advantages of the use of porous materials include capillary flow (which removes the need for equipment for pumping fluids) and lower materials costs. The Whitesides group generated renewed interest in paper-based devices in 2007 with their demonstration of the use of patterned cellulose to simultaneously detect glucose and protein in urine samples [14]. There has been much progress in the field since this initial demonstration [15,16,17], including demonstrations of integrated blood-to-plasma processing in paper-based devices to detect glucose [18], protein [19], and markers of liver function [20].

Previously, we described work to develop a paper-based device for qualitative Phe detection to enable newborn PKU screening in low-resource settings [21]. Our initial work focused on the conversion of a laboratory-based colorimetric assay for Phe into a paper-based format, with dry reagents and a rapid time-to-result of 10 min. The device was demonstrated to have the capability to discriminate between normal and elevated levels of Phe spiked into human plasma samples. Though promising, that device did not include the integrated sample preprocessing that would be necessary for the device to be compatible with finger-stick blood samples at the point of care.

In the work reported here, we detail the further development of a novel paper-based device for Phe monitoring with integrated blood processing. The incorporation of integrated blood processing required a substantial redesign of the previous device [21]. Candidate materials were assessed for the ability to support each reaction, and to accept plasma from the commercially-available plasma separation membrane. The final choice of device material considered both the material-dependent signal and the fluidic compatibility of materials, in order to meet the requirements of the application. The device provided a semi-quantitative colorimetric determination of Phe level in a small (40 μL) sample of whole blood in approximately 8 min. Device performance was characterized with respect to quantitative resolution and reproducibility using an instrument for readout. In addition, the potential use of the device as a monitoring tool of Phe levels for PKU patient therapy was considered and next steps discussed.

**Figure 1:**
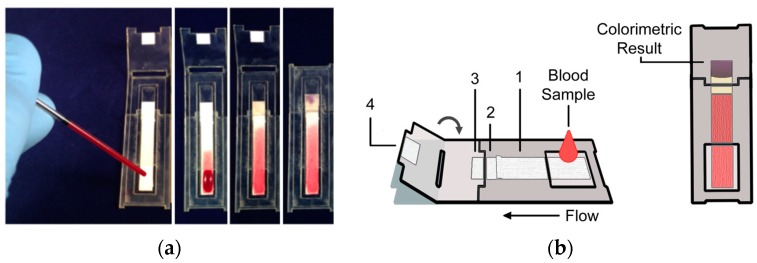
Prototype disposable paper-based device. (**a**) Image series showing different stages of a device run. From left to right, 40 μL of whole blood is applied to the device using a disposable glass capillary and plunger (PTS Diagnostics; left), blood flows into the plasma separation membrane, plasma is collected in the pads immediately downstream and the enzymatic reaction takes place, and after the device is folded closed, the colorimetric reaction takes place and signal develops; (**b**) Schematic of the disposable device. The substrate labeled **1** is the blood to plasma separation membrane, substrates **2** and **3** house the enzymatic reaction, and substrate **4** houses the colorimetric reaction. (Right) The device is folded at ~6 min and then read at ~8 min using an instrument for a semi-quantitative result.

User operation of the disposable device, illustrated in the image series and schematic of Figure 1**,** consists of the following simple steps:

- Load: Add a small volume whole blood sample, 40 μL, to the device sample port.
- Fold: 6 min after sample application fold the card closed and press firmly on the detection region.
- Read: 8 min after sample application read the result using an instrument.

In this work, the whole blood sample was applied to a device using a pipette. However, in the field, a disposable capillary and plunger would provide a simple method for the user to collect 40 μL of whole blood to apply to the device.

## 2. Materials and Methods

*Chemical reactions.* The paper-based microfluidic device uses a well-known set of reactions [21,22,23] for Phe detection from a whole blood sample shown below:

(1) $$\text{Phe}+{\text{NAD}}^{+}\overset{\text{PheDH}}{\to }\text{NADH}+\text{PhePyr}+{\text{NH}}_{4}^{+}$$

(2) $$\text{NBT}+\text{NADH}+\text{mPMS}\to \text{Formazan product}+{\text{NAD}}^{+}$$

NAD^+^ is the oxidized form of nicotinamide adenine dinucleotide, PheDH is the enzyme phenylalanine dehydrogenase, NADH is the reduced form of nicotinamide adenine dinucleotide, PhePyr is phenylalanine pyruvate, NH_4_^+^ is the protonated form of ammonia, NBT is the tetrazolium salt nitroblue tetrazolium, and mPMS is the electron mediator methoxyphenazine methosulfate. NBT was chosen as the tetrazolium salt due to its stability and its conversion into a dark purple formazan dye that can be easily visualized on the white background of the porous substrate [21].

*Fabrication of the paper-based device.* Porous materials were cut to the desired dimensions using a CO_2_ laser cutting system (H-Series, Full Spectrum Laser, Las Vegas, NV, USA), and then assembled into a folding card device (Figure 1). For each test, one side of the Mylar (Tekra, New Berlin, WI, USA) folding card contained a Vivid™ polysulfone plasma separation membrane (Pall Corporation, Port Washington, NY, USA) to separate whole blood into plasma (substrate 1 in Figure 1b), and two glass fiber porous pads (A/E, Pall Corporation, Port Washington, NY, USA) for the enzymatic reaction (substrates 2 and 3 in Figure 1b). The opposite side of the folding card contained a glass fiber pad for the colorimetric reaction (substrate 4 in Figure 1b). Folding the card closed initiated fluid transfer between the glass fiber pads on opposite sides of the folding card. Other materials evaluated included another type of glass fiber (8975, Ahlstrom, Helsinki, Finland) and cellulose (CFSP22300, EMD Millipore, Darmstadt, Germany). Reagents were dried into the porous material pads for storage and rehydration at the time of use. Buffer was applied uniformly to pads by pipetting 10 μL of 220 mM bis-tris propane buffer, pH 9.3, to one 10 μL capacity glass fiber pad (substrate 2 in Figure 1b), and 10 μL of 220 mM bis-tris propane, pH 6.3, to another 10 μL capacity glass fiber pad (substrate 4 in Figure 1b). In addition, 10 μL of 20 mM NAD^+^ (Sigma Aldrich, St. Louis, MO, USA) in deionized water was added to a third 10 μL capacity glass fiber pad (substrate 3 in Figure 1b). The pads were allowed to dry overnight in a desiccator (Secador, Bel-Art Products, Wayne, NJ, USA). The glass fiber pads filled with dry buffer salts were then adhered onto the Mylar card (substrate 2 and 4 in Figure 1b). A 2 μL volume of 40 U/mL PheDH (Sigma Aldrich, St. Louis, MO, USA) was spotted into the center of the downstream edge of the pH 9.3 buffered glass fiber pad (substrate 2 in Figure 1b). A 3 μL volume of a mixture of 1.2 mM NBT (Life Technologies, Eugene OR) and 0.1 mM mPMS (Dojindo Laboratories, Kumamoto, Japan) was spotted into the center of the upstream edge of the pH 6.3 buffered glass fiber pad (substrate 4 in Figure 1b). The spotted pads were allowed to dry for 2 h in a desiccator before use. The plasma membrane was placed on the Mylar card so that there was 1.5 mm of overlap with the upstream edge of the pH 9.3 buffered glass fiber pad (substrate 1 in Figure 1b). The glass fiber pad containing NAD^+^ was placed on top of the downstream edge of the pH 9.3 buffered glass fiber pad so that there was a 1.5 mm overlap (substrate 3 in Figure 1b).

*Enzymatic reaction compatibility with porous substrate.* The cellulose and glass fiber materials described above were cut into circular pads with a capacity of 60 μL using the CO_2_ laser cutting system. Additionally, cellulose was cut using a paper cutter (into squares with a similar capacity). Porous pads were filled to capacity with a mixture of 10 mM NAD^+^ in water and either 0 or 3.8 mg/dL Phe in water, and dried overnight in a desiccator. Dry pads were filled to capacity with 1 U/mL phenylalanine dehydrogenase in 220 mM bis-tris propane (BTP) buffer (pH 9.3), and the enzymatic reaction was performed within the porous material for 3 min. Pads were then centrifuged for 30 s at 16,000 g to recover the reaction fluid. For each reaction, 40 µL of fluid was pipetted into the well of a 384-microwell plate and the absorbance signal at 340 nm was measured 12 min after centrifugation using a plate reader (Ultramark, Bio-Rad Laboratories, Hercules, CA, USA). Eight replicates were performed for each Phe concentration in each substrate.

*Colorimetric reaction compatibility with porous substrates*. Porous material components were cut as described in the previous paragraph. Pads were impregnated with either 0 or 3.8 mg/dL NADH in 220 mM BTP buffer (pH 6.3) and dried overnight in a desiccator. A Mylar folding card contained a dry, reagent-loaded pad on one side of the card, and a virgin pad of the same material on the opposite side. The virgin pad was filled to capacity with a mixture of 0.1 mM mPMS in BTP (pH 6.3) and 1.2 mM NBT in water and the colorimetric reaction initiated when the two pads were placed in contact by folding the card closed. The reaction was performed within the porous material for 3 min and the material was then scanned and analyzed as described in the image acquisition and analysis methods section below. Six replicates were performed for each NADH concentration in each substrate.

*Compatibility of the plasma separation membrane with porous substrates.* The plasma separation membrane was cut to have a capacity of 40 µL, and cellulose and glass fiber materials described above were cut to have a capacity of 30 µL with the CO_2_ laser cutting system. The plasma separation membrane was layered on top of the porous substrate of interest with a 1.5 mm overlap. The assembly was housed within a Mylar folding card to ensure reproducible contact between the two materials. A 40 μL volume of blood was pipetted into the plasma separation membrane and the penetration of plasma into the downstream porous material was imaged after 6 min. Five replicates were performed for each substrate.

*Creation of mock samples in whole human blood.* Blood was collected from two healthy donors and pooled (about 20 mL, one day before the experiment) in accordance with the IRB-approved study protocol. The highest concentration mock sample, was created by diluting 1 part of a concentrated Phe stock solution, 1500 mg/dL Phe (Sigma Aldrich, St. Louis, MO, USA) in deionized water, into 99 parts of pooled normal blood (containing endogenous Phe). Additional mock samples with lower Phe concentrations were created by making three, two-fold serial dilutions with the pooled normal blood as the diluent. As an independent check that the mock samples in whole human blood were in the correct Phe range, blood spots were collected on filter paper and Phe levels determined by mass spectrometry analysis of the blood spot samples (Wisconsin State Laboratory of Hygiene). The Phe levels of the mock samples, as measured by mass spectrometry analysis from blood spots, were 1.1, 2.4, 4.0, 6.3, and 11 mg/dL in increasing order, starting with the pooled normal blood sample, and confirmed that our spiked-in Phe levels were in the correct range. Using an estimate of 1.1 mg/dL for the Phe concentration of the pooled normal blood, the mock samples were calculated to have Phe concentrations of 1.1, 3.0, 4.9, 8.6, and 16 mg/dL in increasing order of Phe concentration. The Phe levels measured by mass spectrometry of blood spot samples were consistently higher than the calculated values of Phe concentration, with an average difference of ~24%. The Phe concentration axis of the calibration curve was set using the calculated Phe concentrations (1.1 to 16 mg/dL). This choice was made for two reasons. First, it has been noted that Phe levels measured from dried blood spot samples using either mass spectrometry or HPLC are significantly lower than Phe levels measured from plasma samples using the amino acid analyzer, between 12% [10] and 26% [24] depending on the study. Second, this choice is the more conservative one in reporting the quantitative resolution values of the device below.

*Assay calibration*. A 40 µL volume of each blood sample (five Phe concentrations including the “normal” containing only endogenous Phe of the pooled normal blood) was spotted onto the upstream region of a plasma separation membrane pad in a multi-test folding card using a multichannel pipette. After 6 min the multi-test folding card was closed such that the glass fiber pads on opposite sides of the previously open folding card made contact. Fluid transfer was aided by the application of firm pressure on the pads. The detection regions of the multi-test folding card were simultaneously scanned after an additional 2 min. Six replicates were performed for the Phe concentration series.

*Image acquisition and analysis methods.* Image data was acquired using a desktop scanner (Perfection V700 Photo, Epson, Nagano, Japan) and analyzed using a custom MATLAB (Natick, MA, USA) program. The colorimetric signal was quantified as follows. For the porous substrate compatibility experiments the signal was calculated as the average grayscale intensity in a circular region of interest within the pad (centered with a diameter of 8 mm) subtracted from the background average grayscale intensity in a control pad with water only. The average final signal intensity was calculated over replicates (N = 6) for each NADH concentration. For the phenylalanine assay, the detection region was defined relative to a fiducial mark on the device, in order to minimize bias and variability in the data extraction procedure. Specifically, the left upstream corner of the detection pad served as the fiducial mark for analysis and the rectangular region of interest (4 mm × 3 mm), was defined to be 2.5 mm to the right and 2.5 mm downstream of that mark. A background signal value was obtained by calculating the average grayscale intensity (N = 5) in the region of interest of devices prepared with all reagents with the exception of the enzyme phenylalanine dehydrogenase. The raw signal value for each Phe concentration of the calibration data set was then obtained by calculating the average grayscale intensity in the region of interest. The final signal intensity for each Phe concentration of the calibration data set was then calculated by subtracting the 8-bit average grayscale intensity for a given Phe concentration from the average background grayscale intensity. The average final signal intensity was calculated over replicates (N = 6) for each Phe concentration. The standard error was calculated and displayed as error bars. The quantitative resolution of the device was estimated using the calibration data over a range of Phe levels. The average standard deviation of points in the range was divided by the slope of the best-fit line through the average data points in that same range (*R*^2^ greater than 0.97).

## 3. Results and Discussion

**Figure 2:**
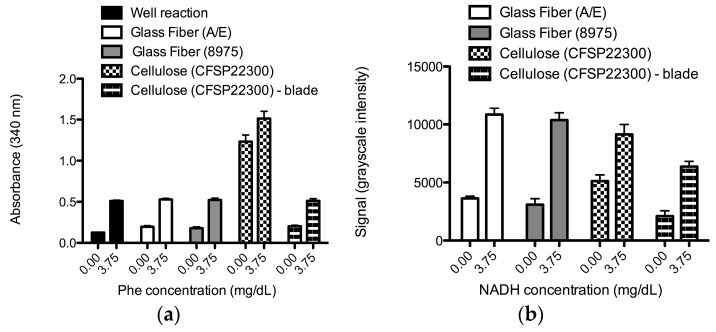
Porous materials compatibility with respect to an ability to support either reaction (1) or (2), separately. (**a**) Average absorbance at 340 nm for enzymatic reactions exposed to glass fiber and cellulose materials (N = 8); (**b**) Compatibility of each porous material with respect to the colorimetric reaction as measured by the signal, average grayscale intensity (N = 6). The error bars represent standard error (only the upper error bars are displayed). Porous materials were cut to the desired sizes using a laser cutting system, with the exception of cellulose, which was also cut using a blade.

The final choice of material for this device was motivated by two critical design constraints: materials compatibility with the two reactions and efficient transfer of plasma from the plasma separation membrane to the downstream porous substrate. The plot of Figure 2a compares NADH production from the enzymatic reaction for candidate porous materials, two different glass fiber materials, and one cellulose material. The results indicate that both of the laser-cut glass fiber materials supported the enzymatic reaction at a level comparable to the reaction performed exclusively in a well. In contrast, the reaction in laser-cut cellulose showed a high background signal that was eliminated when using blade-cut cellulose. The laser cutting process produced a by-product (of the thermal degradation of cellulose) that contributed to the absorbance signal at 340 nm (*i.e.*, an increased absorbance signal from buffer only exposed to laser-cut cellulose *versus* blade-cut cellulose was observed in data not shown here). This contribution to the absorbance signal of laser-cut cellulose could not, by itself, explain the magnitude of the difference in the signals from reactions exposed to laser- *versus* blade-cut cellulose in Figure 2a. It is likely that there was an increased reduction of NAD^+^ to NADH in the laser-cut cellulose compared to the blade-cut cellulose. The plot of Figure 2b compares the signal intensity from the colorimetric reaction for the candidate porous materials. The glass fiber materials produced comparable specific signal for the positive NADH concentration sample above the background signal of the zero NADH control, while use of the cellulose material—laser or blade cut—produced a lower specific signal above the corresponding background signal. Note that the larger colorimetric signals for the laser-cut cellulose compared to the blade-cut cellulose could be explained by an increased reduction of the tetrazolium salt to its corresponding formazan dye in the absence of NADH within the laser-cut cellulose compared to blade-cut cellulose. All of the materials had a relatively large fluid capacity (40, 55, and 56 µL/cm^2^ for A/E glass fiber, 8975 glass fiber, and cellulose, respectively) to enable downstream reactions.

**Figure 3:**
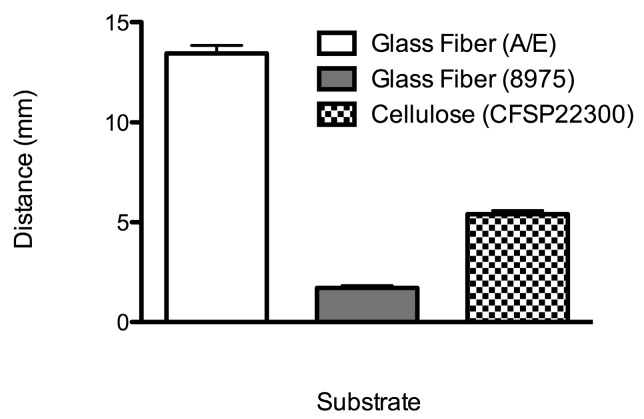
Approximate average distance of plasma wicked into each porous material from whole blood applied to the plasma separation membrane (N = 5). The error bars represent standard error (only the upper error bars are displayed). Plasma from the plasma separation membrane did not flow readily into the larger pore glass fiber (8975) with the fluid front only infiltrating the region of overlap of the two materials.

A second criteria for choosing device materials was efficient transfer of plasma from the plasma separation membrane into the downstream porous component. The relative capillary pressures of a given fluid in any two materials determine the compatibility of these materials with respect to efficient fluid transfer. The plot of Figure 3 presents data for the compatibility of flow with the plasma separation membrane. The plasma separation membrane used in this study showed reliable transfer of plasma into the smaller pore glass fiber and cellulose, but not into the larger pore glass fiber that was used in the previous Phe device [21].

Based on these results, the A/E glass fiber material was chosen as the downstream porous material in our Phe assay device. The disposable device was created by layering porous material components onto an adhesive/Mylar composite substrate. First, blood-to-plasma separation of a 40 μL whole blood sample was achieved using the graded-pore, plasma separation membrane [20,25,26]. Second, the enzymatic reaction was initiated when the processed plasma sample was passively transferred to a set of glass fiber substrates in which NAD^+^, PheDH, and buffer salts had been dried to allow the enzymatic reaction to take place at alkaline pH. Storage of NAD^+^ at acidic pH was required to avoid the production of NADH in the absence of Phe that could lead to a false positive signal [21,27]. Third, the colorimetric reaction at slightly acidic pH was initiated at 6 min after sample application when fluid was actively transferred to a final glass fiber substrate in which NBT, mPMS, and buffer salts had been dried. The choice to incorporate a folding step [28,29] into the device operating protocol has the advantage of keeping the design simple at the expense of requiring a timed user step [21].

**Figure 4:**
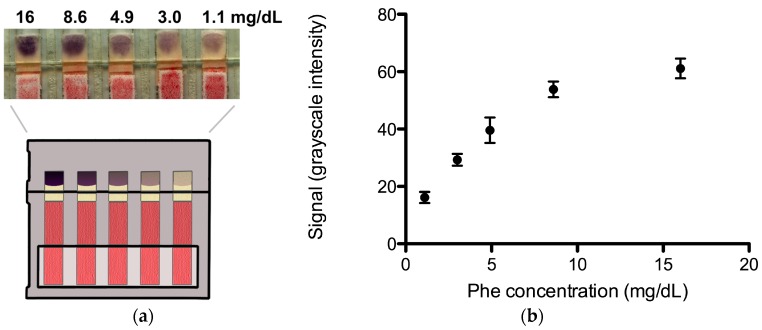
Paper-based whole blood Phe monitor. (**a**) Image data of a concentration series of Phe spiked into whole blood; (**b**) The plot shows the Phe concentration *vs.* signal (mean grayscale intensity) for N = 6 replicates. The error bars represent standard error. See Methods for details on the grayscale intensity scale.

Representative results for a concentration series of Phe in whole human blood—0 to 15 mg/dL of Phe spiked into pooled normal blood to simulate the Phe levels in PKU patients—are shown in Figure 4a. The labels correspond to the calculated concentration of Phe in each of the samples assuming that the pooled normal blood had a concentration of 1.1 mg/dL (see Methods for a detailed discussion). A calibration curve of the average signal extracted from the image data *vs.* blood Phe concentration for six replicates is shown in Figure 4b. The quantitative resolution is 1.4 mg/dL for Phe concentrations between ~1 and 9 mg/dL. The standard error is displayed as error bars for each Phe concentration spanning 1 and 16 mg/dL. The potential utility of measuring blood Phe levels in this range is as follows. Values of 1 to 2 mg/dL are representative of the Phe levels in normal non-PKU persons, values of 2 to 6 mg/dL are representative of the Phe levels in PKU patients who are on target with their therapy [3,4], values of 6 to 9 mg/dL are representative of the Phe levels of PKU patients who are slightly above the target range, and values above 9 mg/dL are representative of the Phe levels of PKU patients who are significantly above the target range. Specific patient actions could be associated with each range. PKU patients with Phe levels in the target range would continue their current therapy regimen, PKU patients with Phe levels that are slightly above the target range would monitor their dietary Phe intake more closely, and PKU patients with Phe levels that are significantly above the target range would monitor their dietary Phe intake more closely and make an appointment to see their physician. The exact ranges for the latter two categories and patient-specific actions would be determined through further discussion with clinicians and PKU patients in light of current therapy and monitoring practices.

This report demonstrates a milestone in the development of a whole blood Phe monitoring device and defines the additional work required to translate this device for use in the field. First, long-term dry reagent storage via vacuum- or freeze-drying is needed to improve reagent shelf life and stability. Second, additional work is needed to improve the robustness of the device. This includes the development of a process control that changes color to indicate a working test and the appropriate time to read the test signal, and on-device calibrators [17]. The robustness of device performance could also be increased by removal of the timed folding step. This step has the potential for user error in timing and in accurate folding. Potential methods for automating this step include dissolvable barriers [30] and expanding mechanical valves [31]. Third, the testing of PKU patient samples in a clinic setting is needed to further validate device performance. And finally, the potential of built-in visual readout using a standalone device warrants further investigation. Specifically, the performance of visual readout should be assessed with respect to accuracy in distinguishing blood Phe levels in distinct ranges of clinical interest. Though visual readout will not provide the level of quantitative resolution or reproducibility achievable by instrument-based readout, if visual readout can meet the minimum performance requirements of the application it would have the advantage of lower cost (no costs for instrument/component manufacturing [16,32]).

## 4. Conclusions

We have demonstrated a disposable, paper-based microfluidic device that uses small volume samples of whole blood to provide semi-quantitative Phe detection when paired with an instrument for colorimetric readout. The device is simple, easy to operate and delivers results in approximately 8 min. The device has the potential to improve the care of PKU patients by allowing them to frequently monitor their Phe levels, and then appropriately adjust nutritional or medication-based therapy in near real-time to improve metabolic control and quality of life.
